# Climate overrides fencing and soil mineral nutrients to affect plant diversity and biomass of alpine grasslands across North Tibet

**DOI:** 10.3389/fpls.2022.1024954

**Published:** 2022-12-08

**Authors:** Chenrui Guo, Karsten Wesche, Mihai Ciprian Mărgărint, Arkadiusz Nowak, Iwona Dembicz, Jianshuang Wu

**Affiliations:** ^1^ Institute of Environment and Sustainable Development in Agriculture, Chinese Academy of Agricultural Sciences, Beijing, China; ^2^ School of Environmental Science and Engineering, Hebei University of Science and Technology, Shijiazhuang, China; ^3^ Department of Botany, Senckenberg Museum of Natural History Görlitz, Görlitz, Germany; ^4^ International Institute (IHI) Zittau, Technische Universität Dresden, Zittau, Germany; ^5^ German Centre for Integrative Biodiversity Research (iDiv) Halle-Jena-Leipzig, Leipzig, Germany; ^6^ Department of Geography, Geography and Geology Faculty, Alexandru Ioan Cuza University of Iaşi, Iaşi, Romania; ^7^ Botanical Garden Center for Biological Diversity Conservation in Powsin, Polish Academy of Sciences, Warsaw, Poland; ^8^ Institute of Biology, University of Opole, Opole, Poland; ^9^ Department of Ecology and Environmental Conservation, Institute of Environmental Biology, Faculty of Biology, University of Warsaw, Warsaw, Poland

**Keywords:** aboveground biomass, grazing exclusion, mineral elements, plant species diversity, soil micronutrient, Northern Tibetan Plateau

## Abstract

**Introduction:**

Overgrazing and warming are thought to be responsible for the loss of species diversity, declined ecosystem productivity and soil nutrient availability of degraded grasslands on the Tibetan Plateau. Mineral elements in soils critically regulate plant individual’s growth, performance, reproduction, and survival. However, it is still unclear whether plant species diversity and biomass production can be improved indirectly via the recovery of mineral element availability at topsoils of degraded grasslands, via grazing exclusion by fencing for years.

**Methods:**

To answer this question, we measured plant species richness, Shannow-Wiener index, aboveground biomass, and mineral element contents of Ca, Cu, Fe, Mg, Mn, Zn, K and P at the top-layer (0 - 10 cm) soils at 15 pairs of fenced vs grazed matched sites from alpine meadows (n = 5), alpine steppes (n = 6), and desert-steppes (n = 4) across North Tibet.

**Results:**

Our results showed that fencing only reduced the Shannon-Wiener index of alpine meadows, and did not alter aboveground biomass, species richness, and soil mineral contents within each grassland type, compared to adjacent open sites grazed by domestic livestock. Aboveground biomass first decreased and then increased along with the gradient of increasing Ca content but did not show any clear relationship with other mineral elements across the three different alpine grassland types. More than 45% of the variance in plant diversity indices and aboveground biomass across North Tibet can be explained by the sum precipitation during plant growing months. Structural equation modelling also confirmed that climatic variables could regulate biomass production directly and indirectly via soil mineral element (Ca) and plant diversity indices.

**Discussion:**

Overall, the community structure and biomass production of alpine grasslands across North Tibet was weakly affected by fencing, compared to the robst climatic control. Therefore, medium-term livestock exclusion by fencing might have limited contribution to the recovery of ecosystem structure and functions of degraded alpine grasslands.

## Introduction

Grasslands cover approximately 40% of the land surface and play a critical role in biodiversity conservation, food security, and climate regulation worldwide (Wang et al., 2010; O'Mara, 2012). Livestock grazing is the most widespread land use to ensure the livelihood security of smallholder pastoralists (Hopping et al., 2018). However, arid grasslands are predicted to increasingly degrade due to global warming and overgrazing at local scales (Gang et al., 2014; Zhang et al., 2018), with consequences of restricted plant growth, declined moisture availability, and reduced soil nutrients (Conant and Paustian, 2002; Wesche et al., 2016; Liu et al., 2019). Thus, grazing exclusion *via* fencing is increasely recommended as a nature-based measure to self-recove of degraded grasslands (Noulekoun et al., 2021; Sun et al., 2021).

Alpine grasslands are vulnerable and sensitive to climate change and land-use shifts. A programme entitled the ‘Returning Grazing Land to Grassland’, jointly financed by local authorities and the central government, was implemented in 2003 and has lasted for two decades to recover degraded grasslands in Mainland China. Approximately 57,600 km^2^ of degraded alpine grasslands in North Tibet have been fenced and excluded from livestock grazing under this programme (Yu et al., 2016). Recently studies revealed that aboveground biomass (Zhao et al., 2019; Liu et al., 2020a), plant species diversity (Zhu et al., 2016; Li et al., 2018), and soil nutrient availability of organic carbon, N, P, Fe, Mn, and Cu (Wu et al., 2010; Li et al., 2011; Sun et al., 2020) can be improved due to fencing on the Tibetan Plateau. However, others argued that fencing has neutral or even negative effects on plant diversity indices (Yao et al., 2019; Liu et al., 2020b), biomass production (Wu et al., 2017b; Sun et al., 2020) and soil nutrient content of N, P, Cu, Mn, and Zn) (Lu et al., 2015b; Jiao et al., 2016). Therefor, it is still under debate whether and how fencing can recover degdraded alpine grassland on the Tibetan Plateau.

In addition to N and P, grassland productivity can also be co-limited by other mineral elements. For example, Fay et al. (2015) and Radujkovic et al. (2021) have pointed out that less-studied nutrients, such as Ca, Mg and K, and trace elements, such as Fe, Cu, Mn and Zn, have considerable influences on grassland plant’s performance and survival, as some of them can enhance enzymatic reactions and are critical in protein synthesis. For example, Ca at top soils can indirectly influence alpine plant growth by regulating their tolerance to low temperatures and their tissue palatability to avoid herbivores uptaking (Fageria et al., 2002; Fu et al., 2006). The effects of trace elements on crop plants have been well explored; however, it is unexplored whether they can be affected due to shited land-use from being grazed to fenced. Moreover, little is known about whether the potential changesin soil mineral elements caused by land-use shift can be cascade to regulate plant community structure and prodution of degraded grasslands.

In this study, we conducted a multisite survey to compare aboveground biomass, species richness Shannon-Wiener index, and mineral element contents of Ca, Cu, Fe, Mg, Mn, Zn, K and P of the topsoils at grazed vs fenced matched sites across alpine meadows (AM), alpine steppes (AS) and desert-steppes (DS) in North Tibet. Specifically, we aimed to answer the following questions: (1) has medium-term grazing exclusion by fencing altered plant community structure and mineral element contents? And (2) how do soil mineral elements and climate factors, including precipitation and temperature during plant growing months, regulate plant diversity and productivity within and across the community levels? Here, we hypothesize that (1) aboveground biomass and plant diversity are joinly affected by soil mineral elements and local climatic conditions while rarely by livestock exclusion by fencing; and (2) the effects of fencing and soil mineral elements on plant communities specifically differ among alpine grassland types.

## Materials and methods

### Study area

Locally known as Changtang, North Tibet is the most traditional and vastest pastoral region within the Tibetan Autonomous Region, China. It covers about 480,000 km^2^ of alpine grasslands (Wu et al., 2013) and has the largest nature reserve in China for conserving Tibetan antelopes, wild yaks and kiangs. Alpine grasslands in North Tibet have been overgrazed by domestic yaks, sheep, and goats for decades. The total number of livestock reached 23,490,000 heads in 2010, which was about 89.4% higher than the theoretical capacity of all available alpine grasslands in the developing Tibet (Yu et al., 2012).

**Figure 1:**
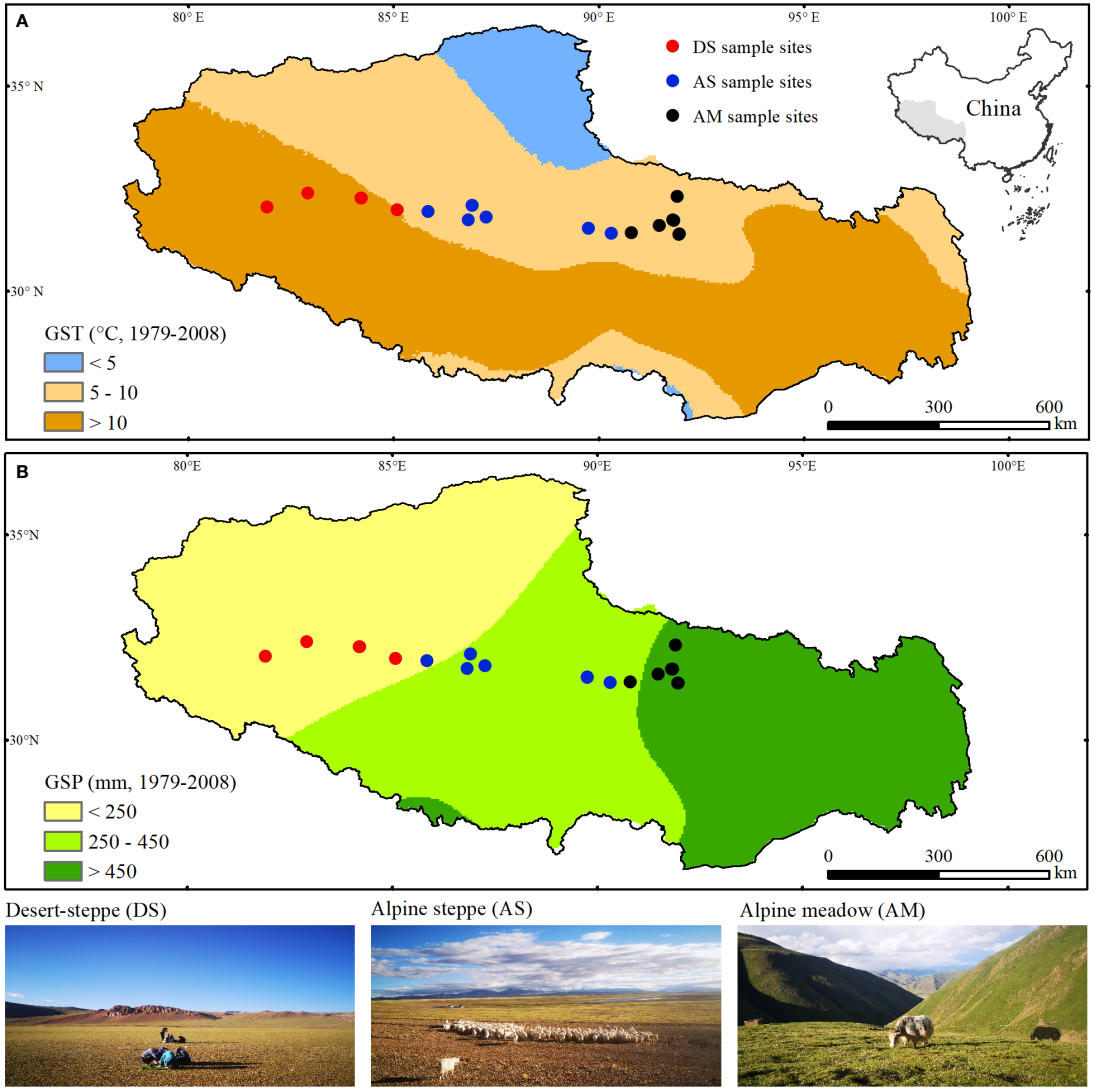
Sampling sites across North Tibet and climate conditions of the entire Tibetan Autonomous Region of China. Panel **(A)** shows mean temperature (GST) and **(B)** sum precipitation (GSP) during the plant gowing seasons from 1979 to 2008. Landscape pictures are also given for desert-steppe (DS), alpine steppe (AS), and alpine meadow (AM) along climatic gradients.

Mean annual precipitation in North Tibet decreases westwards from more than 450 mm to less than 250 mm, while mean annual temperature increases from −2°C to 1.2°C from east to west (Wu et al., 2017b). In the last century, the Tibetan Plateau experienced fast warming at a rate of 0.3°C per decade, which is about twice the global average (Qiu, 2008). Meanwhile, precipitation changed unevenly across Tibet (Liu et al., 2008), indicating warming-drying and warming-wetting co-exist on this plateau. Daily air temperatures are always higher than 5°C since May (**Figure 1A**). Meanwhile, approximately 65% to 85% of the yearly precipitation falls between May and September (**Figure 1B**). So, the period from May to September was defined as the plant growing season and widely used in recent research on alpine vegetation (Ma et al., 2010; Wang et al., 2013).

Most grassland plants in North Tibet are annuals or biennials and they generally sprout in early May, reach their maximum height/coverage in mid-August, and then senescence in late September. This is also why the period from May to September has increasingly termed the plant growing season in recent ecological studies on the Tibetan Plateau (Shen et al., 2015a; Shen et al., 2015b). In North Tibet, zonal vegetation shifts from alpine meadows dominated by *Kobresia pygmaea* to alpine steppes dominated by *Stipa purpurea* and finally to desert-steppes co-dominated by *S. glareosa* and *S. subsessiliflora* in the west (**Figure 1**).Warming and overgrazing have resulted in the degradation of 153,000 km^2^ of alpine grasslands in this region (Yu et al., 2016). Between 2003 and 2012, fences were built on approximately 57,600 km^2^ of severely degraded pastures to exclude livestock grazing for self-recovery.

### Field survey and measurement

To examine the effects of grazing exclusion on plant species diversity, soil mineral nutrients, and aboveground biomass, 15 pairs of grazed vs fenced matched sites were sampled in the summer of 2012, at intervals of 50 - 80 km, from three alpine grassland types - alpine meadows (AM, n = 5), alpine steppes (AS, n = 6), and to desert-steppes (DS, n = 4, **Figure 1** and **Table 1**). Local authorities and experienced herders proposed the site locations as they knew well about the extent and degree of degradation before fencing.

**Table 1:** Site locations, climate conditions, aboveground biomass (AGB), species richness, livestock composition, and average stocking rate of herd households around sampling sites are summarized at the county level and within each of the three grassland types, alpine meadows (AM), alpine steppes (AS), and desert-steppes (DS) across North Tibet.

Grasslands	AM		AS		DS	
County	Nagqu	Amdo	Nyima	Baingoin	Gêrzê	Gê’gyai
Sites	3	2	3	3	2	2
Longitude (°E)	91.48-91.97	91.83-91.91	86.91-89.75	90.31-90.80	84.20-85.08	81.91-86.82
Latitude (°N)	31.37-31.62	31.71-32.30	31.52-32.08	31.39-31.41	31.97-32.26	31.73-32.38
Altitude (m)	4528-4540	4620-4689	4531-4604	4590-4608	4477-4559	4437-4658
GSP (mm)	383-400	396-422	258-334	349-365	178-185	89-133
GST(°C)	7.7-8.3	7.3-7.9	7.4-8.4	7.7-7.9	10.1-10.6	11.1-11.6
AGB (g·m^-2^)	74.0 ± 9.2	94.3 ± 9.2	30.7 ± 2.2	34.8 ± 4.2	15.9 ± 1.2	11.5 ± 2.2
Species richness (n·per 0.25 m^-2^)	7.3 ± 0.4	11.3 ± 0.7	4.8 ± 0.5	9.1 ± 0.4	4.6 ± 0.3	4.4 ± 0.6
Yak (%)	65	40.1	8.3	19	11.8	3.5
Sheep and goat (%)	34.2	59	91.2	80.5	88	96.3
Average stocking rate (heads·ha^-1^)	0.56	0.12	0.15	0.39	0.05	0.16

Mean values ± standard error are given for AGB and species richness. GST and GSP are abbreviations for average temperature and sum precipitation, respectively, during the plant growing season.

In this study, the fenced plots have been excluded from livestock grazing all year round since 2006. A grazed plot was randomly selected 1-2 km from each fenced plot. The principal livestock in grazed plots are yaks, sheep and goats (**Table 1**). The actual average stocking rate at grazed sites ranges from 0.05 heads•ha^-1^ to 0.56 heads•ha^-1^(**Table 1**). Each pair of fenced and grazed sites was chosen to match as similarly as possible concerning terrain, soil, and climate conditions. Thus, examining the effects of land-use shift on plant diversity, soil nutrients, and biomass production make sense at local scales. However, land-use change is likely to interact with climate and edaphic conditions at the regional scale.

It is impossible for local herders who based nearby the sampling sites to accurately recall the information of livestock activity (timing, intensity and frequency) ten years ago. To infer the stocking rate at the household level, we conducted face-to-face interviews with the heads of herd household nearby our sampling sites in the winter of 2019. We investigated and collected their data of livestock composition and available grasslands (not fenced) at the household level. Meanwhile, we also collected the livestock numbers at the year end from the the statistical yearbook of 2019. Robst linear relationships were found for stocking rates at the county and household levels for each of the three alpine grassland types (**Figure S1**). Using these linear models, we estimated the stock rates of herder families based nearby our sampling sites, for further analyses.

At each fenced or grazed plot, five quadrats of 0.5 m × 0.5 m were laid at 20 m intervals along a random sampling line within a flat area. All plant species were identified and recorded with their names. Plant coverage by species was visually estimated. The heights of all species occurring within each quadrat were measured with a rule. We harvested all plant materials with scissors at the soil surface and stored them in separate envelopes during the field campaign. Plant materials by species were oven-dried at 65 ℃ for 48h to a consistent weight to estimate aboveground biomass and used for calculating diversity indices. A soil block of 25 cm × 25 cm × 10 cm (length × width × depth) was sampled in the center of each quadrat after biomass harvest for further chemical analyses.

### Data management and processing

In each quadrat, species richness (SR) was determined as all the number of vascular plants. Species’ relative coverage (C_r_), height (H_r_) and frequency (F_r_) were measured to calculate the Shannon-Wiener index (Eqns. (1) – (3) as did (Wu et al., 2012). Thirty sampling circles of 0.1 m^2^ were randomly thrown at each fenced and grazed plot, and the number of occurrences for each species (F_i_) was recorded to calculate F_r_.


(1) $$IV=\left(C_{r}+H_{r}+F_{r}\right)/3$$

(2) $$P_{i}=IV_{i}/\sum_{i=1}^{SR}IV_{i}$$

(3) $$\text{Shannon}-\text{Wiener index}=\;-\sum_{i=1}^{SR}P_{i}lnP_{i}$$

where C_r_ is the cover fraction of a given species to sum cover of all species in the quadrat; F_r_ is the frequency percentage of a given species to sum occurrences of all species in the 30 sampling circles; H_r_ is the height rato of a given species to the average height of all species in the quadrat; IV and P, respectively, are the importance value and relative dominance of a given sepcies.

Soil samples were air dried, sieved through a 2-mm sieve to remove stones and roots, and digested by nitric acid, hydrofluoric acid and perchloric acid (3:3:1) to analyze the elements of Ca, Cu, Fe, Mg, Mn, Zn, K and P at the 0-10cm soils using inductively coupled plasma atomic emission spectrometry (ICP-AES) (Silva et al., 2021).

Daily precipitation and temperature of all stations in the Tibetan Autonomous Region were downloaded from the China Meteorological Data Service Center (CMDC, http://data.cma.cn). Monthly raster surfaces of temperature and precipitation were generated with ANUSPLIN 4.3 (Hutchinson, 2004) at a spatial resolution of 1 km × 1 km, then used to calculate the sum precipitation and mean temperature during the plant growing season (noted as GSP and GST, respectively). Climate information for each sampling site was extracted according to its geographic coordinates in ArcGIS 10.2.

### Statistical analysis

First, the differences aboveground biomass, Shannon-Wiener index and species richness, and mineral element contents inside and outside fences across North Tibet and within each grassland type were first examined by t-tests. After the Shapiro-Wilk’s test for data normality and(Bartlett-test for homoscedasticity, we used two-way ANOVA with Tukey’s HSD test to disentangle the effects of fencing, grassland types and their interactions on plant diversity indices and minderal element contents at top soils.

Then, bivariate regressions, including linear and quadratic models, were performed to examine how plant community characteristics vary along with the gradients of mineral element contents at topsoils. Person’s correlation between climate variables (GSP and GST), plant community, and soil variables was performed to examine the collinearity problems among all the responsible and explanatory variables.

Next, we decomposed the relative contribution of each environmental variable (climate and soil) to changes in plant community regimes with multiple linear models at two different spatical scales first across the entire North Tibet and then within each of the three grassland types. The best-fitted models were picked out with the *AICcmodavg* package with a backward simplification approach according to the corrected Akaike’s information criterion (AICc) (Sugiura, 1978).We finally calculated the proportion of the variance explained by each significant variable in the best-fitted model as its effect size (Eta squared, η^2^).

Last, structural equation models (SEMs) using the *lavaan* package (Rosseel, 2012) were constructed to examine the direct and indirect causal links between climate variables, plant community indices, and soil mineral elements. The chi-square (χ^2^) *p* > 0.05 and standardized root mean square residual (SRMR) ≤ 0.05 were accepted as good fitness (Fan et al., 2016). All tests were evaluated at *P* ≤ 0.05 to determine if there was a significant difference. All the data analyses and visualization of this study were conducted with R 4.1.2 (R CoreTeam, 2017).

## Results

### Plant community and contents of soil mineral elements

**Figure 2:**
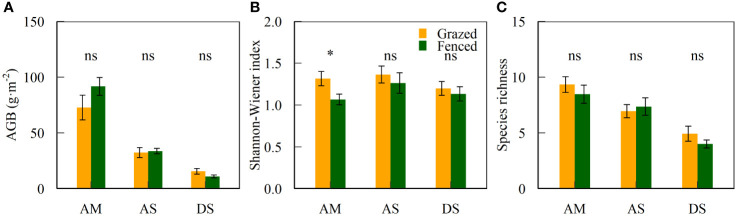
Mean ± standard error of vegetation variables (AGB, aboveground biomass, Shannon-Wiener index, and species richness) inside and outside fences. ^*^ and ns indicates significant (*P* < 0.05) and non-significant differences (*P* > 0.05) between fenced and grazed plots, which is also applicable to **Figure 3**. Abbreviations are the same as in **Table 1**.

Grazing exclusion by fencing did not alter plant diversity and biomass of alpine grasslands in North Tibet (**Table S1**). Aboveground biomass and species richness did not differ between fenced and grazed plots within the three grassland types (*P* > 0.05, **Figure 2**). In alpine meadows, the Shannon-Wiener index in fenced plots was lower than in grazed plots (*P* < 0.05, **Figure 2B**). Aboveground biomass and species richness varied among alpine grassland types (*P* < 0.01, **Table 2**). The interaction of grazing exclusion and grassland types was not significant either (*P* > 0.05, **Table 2**).

**Table 2:** Summary of the two-way ANOVA, in which the effects of land-uses (fencing vs grazing), grassland types (alpine meadows, alpine steppes, and desert-steppes), and their interactions (Land-use × Grassland types) on aboveground biomass, Shannon-Wiener index, species richness, and mineral element contents of Ca, Cu, Fe, Mg, Mn, Zn, K and P at topsoils alpine grasslands in North Tibet.

	Land uses			Grassland types			Land-uses × Grassland types		
	*Df*	*F*	*P*	*Df*	*F*	*P*	*Df*	*F*	*P*
Aboveground biomass	1	0.83	0.365	2	116.77	< 0.001	2	2.99	0.056
Shannon-Wiener index	1	3.18	0.078	2	1.44	0.242	2	0.50	0.609
Species richness	1	0.57	0.452	2	20.93	< 0.001	2	0.46	0.636
Ca	1	0.04	0.735	2	23.29	< 0.001	2	0.41	0.936
Cu	1	0.03	0.876	2	0.27	0.767	2	0.10	0.906
Fe	1	1.04	0.311	2	11.08	< 0.001	2	1.98	0.144
Mg	1	1.14	0.289	2	0.34	0.716	2	1.73	0.183
Mn	1	<0.01	0.950	2	5.28	0.007	2	0.98	0.378
Zn	1	0.03	0.860	2	2.84	0.064	2	0.73	0.484
K	1	0.09	0.771	2	2.13	0.125	2	3.35	0.040
P	1	0.17	0.682	2	1.55	0.217	2	0.86	0.426

**Figure 3:**
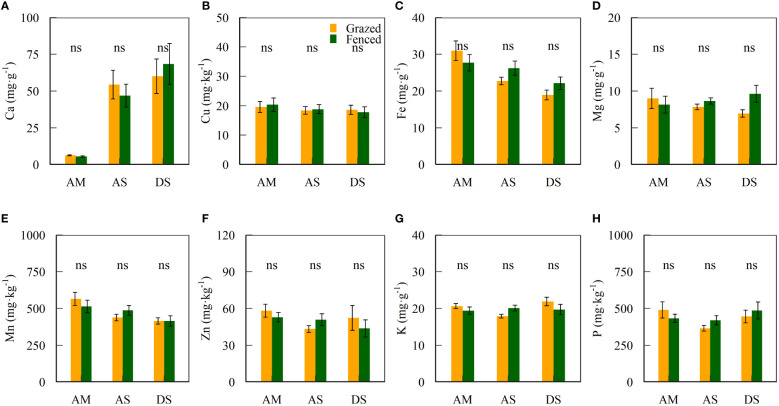
Mean ± standard error of soil mineral elements **(A–H)** inside and outside fences.

The contents of soil mineral elements were not significantly different between fenced and grazed sites (**Table S1** and **Figure 3**). Significant differences were found in Ca, Fe and Mn contents of topsoils between grassland types (*P* < 0.05, **Table 2**). The interaction of fencing and community types only showed significant effects on variance in the K content at top soils (*P* < 0.05, **Table 2**).

### The relationships between plant community properties and environmental factors

Plant community properties were significantly correlated with climate variables (**Figure S2**). Aboveground biomass and species richness were closely related with GSP and GST, with absolute coefficient values being higher than 0.5. Aboveground biomass and species richness were also closely correlated with the Ca content inside and outside fences (**Figures S2**, **S3A**, **S5A**) while closely linked to Fe content in grazed plots (**Figures S3C**, **S5C**). Aboveground biomass and Shannon-Wiener index were closely correlated with K content only in grazed plots (**Figures S3G**, **Figure S4G**).

### Effects of climate variables, soil mineral elements and plant diversity on grassland productivity

GSP alone explained 68.2% of the total variance of aboveground biomass across North Tibet, followed by Ca, which accounted for 5% of its variance. The content of Ca at top soils alone explained 14.1% of the aboveground biomass of alpine steppes. GST explained 5.8% of the total variance of the Shannon-wiener index across different alpine grassland types in North Tibet. GSP explained most of the total variance of species richness across all the three alpine grassland types in North Tibet (45.3%), and also in AM (31.2%), AS (60.1%) and DS (15.0%).

**Figure 4:**
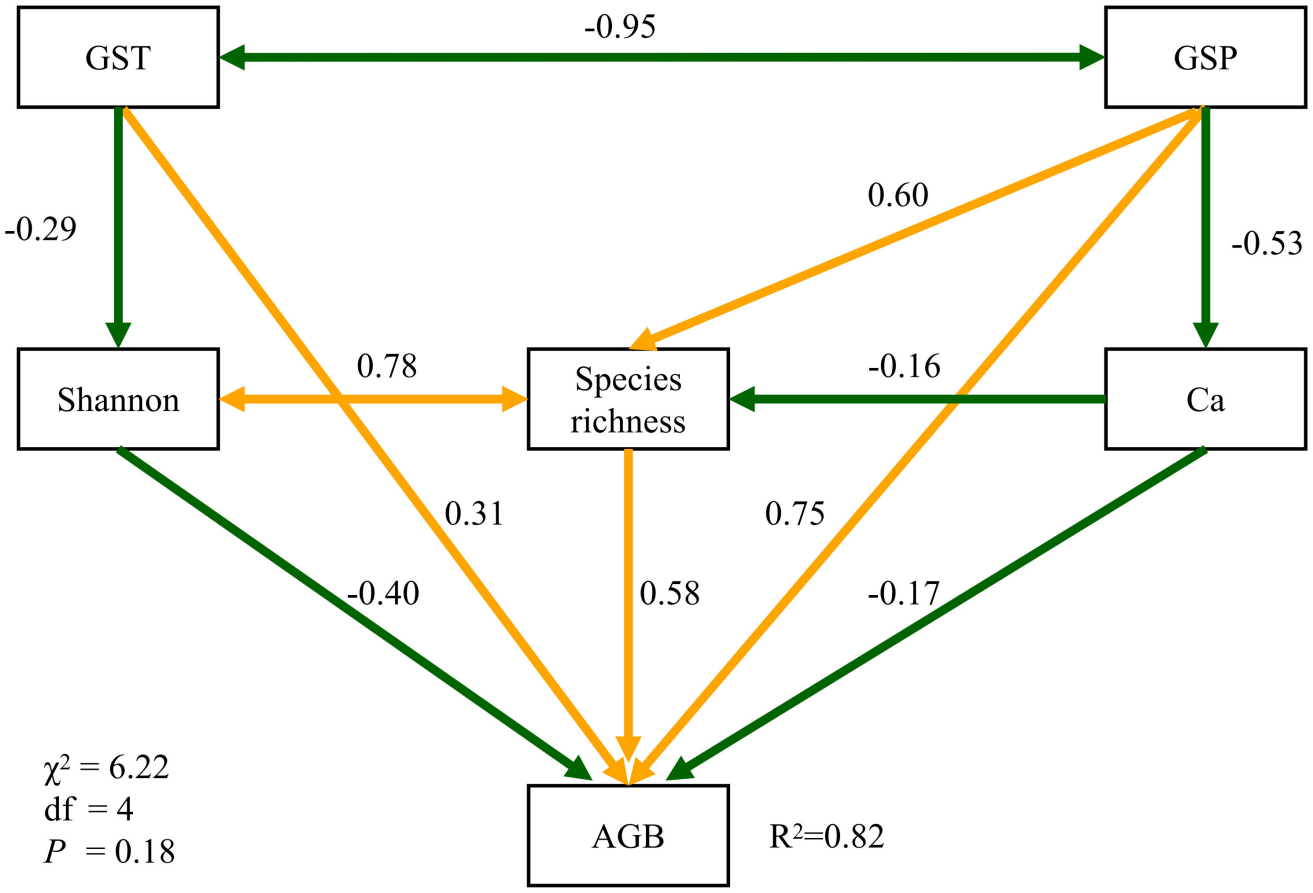
Structural equation models. Dark-green and orange arrows, respectively, indicate the negative and positive associations. All paths are significant at the 0.05 level.

In the structural equation model (**Figure 4**), GSP and GST significantly affected aboveground biomass through standardized direct pathways of 0.75 and 0.31 in strength. GSP also had indirect positive effects on aboveground biomnass *via* species richness (0.35) and the Ca content (0.09). The influences from GSP, GST, Shannon index, species richness and soil Ca explained about 82% of the total variance of aboveground biomass in North Tibet (**Figure 4**). The standardized path strength of GSP affecting plant biomass indirectly *via* soil Ca content was 0.14, when the covariance between Shannon-Wiener index and species richness was excluded (**Figures S6**, **S7**).

## Discussion

### Effects of fencing on plant community regimes

In this study, we selected plant aboveground biomass and diversity at the community level to assess the effect of land use shifts from grazing by livestock to fencing for excluding herbivory on degraded alpine grasslands in North Tibet. Our results showed that six years of grazing exclusion by fencing had a common effect on plant biomass and diversity in alpine grasslands in North Tibet (**Figure 2**). Specifically, such a six-year grazing exclusion by fencing negatively impacted biodiversity in alpine meadows. This result is partially consistent with previous studies on the Tibetan Plateau. For example, medium-term fencing (< 8 years) did not alter either species richness or Shannon-Wiener index (Yan and Lu, 2015; Zhang et al., 2021) but significantly enhanced aboveground biomass of alpine grasslands (Yan and Lu, 2015; Liu et al., 2020a; Zhang et al., 2021). The inconsistent results may be due to the differences in grazing intensity, enclosure duration, original vegetation and soil conditions, and climate background.

First, the climate in Northern Tibet is relatively harsh, with low temperatures at high elevations. Snow or hail often occurs even during the plant-growing months. Low temperatures may negatively affect plants’ uptake of water or nutrients from top soils, and consequently limit the recovery of plant community structure and functions, termed as plant diversity indices and aboveground biomass (Wang et al., 2013). Additionally, the loss of plant diversity in higher productive meadows may be due to increased competition among plant individuals for canopy resources (Wu et al., 2009). More competitive plant species can become more dominant when less competitive plants are restrained after removing the livestock grazing disturbance (Xiong et al., 2016). These results imply that medium-term fencing may have limited and negative influence on plant biomass production and diversity of degraded alpine grasslands at the community level across North Tibet.

### Effects of fencing on soil mineral elements

Mineral elements are crucial in regulating the synthesis of macromolecules and maintaining physiological functions as constituents of enzymes and chlorophyll for plants (Broadley et al., 2012), although they are not required equally in amount as P and N. However, the six-year fencing of this study did not significantly alter the mineral element contents at top soils of degraded alpine grasslands in North Tibet (**Figure 3**). This result is partially similar to that in Li et al. (2011), who found 8-year livestock exclusion did not change the contents of Ca, Mg and Zn at topsoils of the temperate grasslands in Inner Mongolia. Jiao et al. (2016) also found that 8-year fencing did not significantly alter the contents of soil Cu, Mn and Zn, but increased the content of soil Fe of desert-steppes and alpine meadows in Gansu Province, China. Similarly, (Gebregergs et al., 2019) found that 5-year grazing exclusion by fencing did not significantly change the contents of Ca and Mg of semi-arid grassland soils in North Ethiopia.

Several reasons may explain the stable contents of soil mineral elements. First, mineral elements in top soils are mainly derived from long-historical natural processes (such as rock weathering and mineral formation) and influenced by parent materials, pH and organic matter. Six years of fencing may be too short to alter the mineral nutrient contents at top soils (Lu et al., 2015b). Second, the soils of Tibetan alpine grasslands are typically alkaline with relatively high pH values (Lu et al., 2015a), indicating that mineral elements can be easily fixed as insoluble or less-soluble compounds. In addition, fencing may not significantly alter the activity of soil enzymes associated with mineral element cycling (such as β-glucosidase, urase and phosphatase) (Shi et al., 2013; Du et al., 2020) in degraded alpine grasslands, which may limit organic matter decomposition and mineralization. Therefore, fencing has limited influences on the soil mineral nutrients of degraded alpine grasslands in North Tibet.

### Effects of climate and soil mineral nutrients on plant community

Results from multiple and structural equation models demonstrated that precipitation during the plant growing season plays the most prominent role in regulating biomass production of alpine grasslands in North Tibet directly or indirectly through soil Ca and plant diversity indices (**Table 3** and **Figure 4**). These results were consistent with previous studies on alpine grasslands the Tibetan Plateau (Yang et al., 2010; Wu et al., 2014; Wu et al., 2019) and temperate grasslands in other arid and semi-arid places (Ippolito et al., 2010; Cregger et al., 2014). It is likely that precipitation and micronutrients, particularly Zn and Fe, regulate grassland biomass production worldwide (Radujkovic et al., 2021).

Variations in precipitation and temperature may alter the distribution and dynamics of water availability (Davidson and Janssens, 2006) and thus affect soil biochemical conditions and vegetation growth (Nielsen and Ball, 2015). In this case, alpine grasslands’ soil nutrients and plant biomass production were primarily driven by the growing season precipitation rather than grazing exclusion. Therefore, the potential shifts of climate conditions in North Tibet and the specific responses of different grassland types to climate change should be well-considered when making policies for alpine grassland conservation and restoration (Gao et al., 2014; Duan et al., 2021).

The impact of mineral elements on aboveground biomass might become more critical than climatic variables within specific alpine grassland types (**Table 3**). Previous studies also highlighted that the impacts of climate on vegetation and soils varied among different habitats on the Tibetan Plateau (Wang and Wesche, 2016; Wang et al., 2017). As climatic variables are less variable within a given habitat, soil nutrient heterogeneity might strongly influence the variance in plant community structure and productivity at small scales. We found that the Ca strongly influences plant biomass production and explained a considerable fraction of biomass variance, particularly in alpine steppes (**Table 3** and **Figure 4**). Ca plays a crucial role in maintaining cell structure and improving the tolerance of alpine plants to low temperatures (Hepler, 2005; Fu et al., 2006). Therefore, much closer attention should be paid to those undervalued mineral elements (both in soil and plant tissue) regulating plant species’ survival and performance in harsh habitats.

**Table 3:** Effects of climate regimes and soil mineral elements on aboveground biomass (AGB), Shannon-Wiener index, plant species richness of alpine grasslands across North Tibet and at the grassland type level (AM, alpine meadow; AS, alpine steppe; DS, desert-steppe).

Explanatory variable	Northern Tibet					AM				AS					DS			
	Estimate	F		*P*	η^2^	Estimate	F	*P*	η^2^	Estimate	F		*P*	η^2^	Estimate	F	*P*	η^2^
AGB		R^2^ = 80.44					R^2^ = 35.85				R^2^ = 30.7					R^2 =^ 72.11		
GSP	1.71	289.48	<0.01		68.24	-12.23	3.91	0.06	11.39									
GST	2.94	7.28	0.01		1.72	21.44	0.64	0.43	1.87	11.66	0.24	0.62		0.59	2.2	33.53	<0.01	44.53
Richness	0.28	7.96	0.01		1.88	7.19	1.94	0.18	5.66	1.9	2.38	0.13		5.69	-4.02	14.99	<0.01	19.91
Ca	-0.35	21.27	<0.01		5.01	-0.3	3.47	0.08	10.12	-0.19	5.88	0.02		14.05				
Cu															0.35	2.50	0.13	3.32
Fe	1.59	0.73	0.39		0.17	-2.36	0.92	0.35	2.67	0.85	0.44	0.51		1.05	0.53	0.01	0.93	0.01
Mg	-0.71	8.00	0.01		1.88	2.09	0.04	0.85	0.11	-3.54	3.90	0.06		9.33	-1.08	3.27	0.09	4.34
Mn																		
Zn	0.26	2.72	0.10		0.64													
K	-0.53	3.79	0.05		0.89													
P						0.08	1.38	0.25	4.02									
Shannon-Wiener index		R^2^ = 0.16					R^2^ = 0.47				R^2^ = 0.78					R^2^ = 0.41		
GSP						0.01	2.28	0.14	4.84	0.01	81.18	<0.01		62.77	0.02	2.52	0.13	7.09
GST	-1.37	5.88	0.02		5.75										0.84	1.79	0.20	5.03
Ca	0.22	1.83	0.18		1.79	0.14	16.26	<0.01	34.46									
Cu										0.01	2.50	0.12		1.93				
Fe						0.03	0.12	0.73	0.25									
Mg						-0.06	3.52	0.07	7.46									
Mn	-0.56	0.74	0.39		0.72					0	0.91	0.35		0.70	-0.01	3.30	0.08	9.29
Zn										-0.02	13.21	<0.01		10.22	-0.01	3.43	0.08	9.65
K	0.4	3.39	0.07		3.32					-0.31	2.54	0.12		1.96				
P	0.39	4.39	0.04		4.30										-0.01	3.52	0.07	9.89
Species richness		R^2^ = 0.48					R^2^ = 0.75				R^2^ = 0.77					R^2^ = 0.41		
GSP	0.8	76.11	<0.01		45.29	0.23	27.10	<0.01	31.20	0.07	73.90	<0.01		60.11	-0.01	5.30	0.03	14.99
GST						-3.5	2.29	0.14	2.63	1.52	0.36	0.55		0.29				
Ca						0.57	12.19	<0.01	14.04						0.06	0.18	0.68	0.50
Cu										0.11	0.93	0.34		0.76				
Fe															0.23	0.60	0.45	1.71
Mg										-0.29	5.20	0.03		4.23				
Mn	-0.45	0.58	0.45		0.34	0.02	3.85	0.06	4.44	0.02	4.79	0.04		3.90	-0.01	2.63	0.12	7.43
Zn						-0.11	8.41	0.01	9.69	-0.15	9.76	<0.01		7.94				
K						0.47	7.04	0.01	8.10						0.5	3.28	0.09	9.27
P	0.35	3.36	0.07		2.00	-0.01	3.98	0.06	4.58						-0.01	3.39	0.08	9.58

Statistics of F, P, and η^2^ were shown and estimated from the most-fitted multiple linear models. Here, the value of η^2^ means the percentage of the sum squares explained by a given potential explanator

In our study, climate variables also interact with plant community diversity to control plant biomass (**Figures 4**, **S5**, **S6**), although the way of climate *via* Shannon-Wiener index was not significant enough to impact aboveground biomass. These findings also agree with (Klanderud, 2010) that the response of natural grasslands to climate change and grazing disturbance does not necessarily follow a simple linear or unimodal trajectory. More explicit exploration of the relationships between abiotic and biotic variables should be examined first, especially in less productivity communities (Erfanzadeh et al., 2015; Lehnert et al., 2016; Calvo et al., 2021).Therefore, a better comprehension of the response of alpine grassland plants to climate change and human interference is needed.

Our study indicates that grazing exclusion by fencing is limited to restoring vegetation and soil mineral properties in studied alpine grasslands, possibly due to the harsh environment and relatively low stocking rate (**Table 1**). However, the effectiveness of fencing depends on the historical grazing intensities (Wu et al., 2017a). Our study only selected sampling sites in the main counties of different grassland types and failed to consider the actual stocking rate when designing the field survey method. In addition, we did not examine other soil properties (such as pH) that are highly correlated with the content of mineral elements, which may limit better interpretation of the variations in soil mineral elements.

## Conclusion

Plant biomass and diversity of alpine grasslands in North Tibet are weakly affected by grazing exclusion by fencing alone and mainly regulated by growing season precipitation and soil mineral elements at topsoils. However, the effects of medium-term fencing and soil mineral elements on plant communities were more pronounced at the grassland level. Grassland management policies need to be explicitly improved for each grassland type. It is also urgent to uncover the mechanisms of how mineral nutrients regulate alpine grassland productivity under changing climate and shifting management in further studies.

## Data availability statement

The original contributions presented in the study are included in the article/**Supplementary Material**. Further inquiries can be directed to the corresponding author.

## Author contributions

JW conceived the research idea, led the field surveys, and performed the chemical analysis. CG analysed the data. CG wrote the first draft under JW’s supervision. KW, MM, AN, ID and JW interpreted the results, revised the text, and proofread the whole manuscript. Jürgen Dengler provided valuable comments on this article. All authors contributed to the article and approved the submitted version.
