# Supplementary material for: Climate overrides fencing and soil mineral nutrients to affect plant diversity and biomass of alpine grasslands across North Tibet
Source: Front Plant Sci. 2022 Dec 8;13:1024954. doi: 10.3389/fpls.2022.1024954 (PMC9773210; doi:10.3389/fpls.2022.1024954)
Supplement: Supplementary file 1 [file DataSheet_1.docx]

Supplementary Material

**Table S1** Comparisons of plant community characteristics, including aboveground biomass, Shannon-Wiener index and species richness, and the mineral element contents of Ca, Cu, Fe, Mg, Mn, Zn, K and at topsois inside and outside fences. All differences were not significant (*P* > 0.05).

| Plots | Aboveground biomass (g·m^-2^) | Shannon-Wiener index | Species richness | Ca  (mg·g^-1^) | Cu  (mg·kg^-1^) | Fe  (mg·g^-1^) | Mg  (mg·g^-1^) | Mn  (mg·kg^-1^) | Zn  (mg·g^-1^) | K  (mg·g^-1^) | P  (mg·kg^-1^) |
| --- | --- | --- | --- | --- | --- | --- | --- | --- | --- | --- | --- |
| Grazed | 40.2±5.2 | 1.30±0.05 | 7.1±0.5 | 40.7±6.1 | 18.8±0.9 | 24.2±1.2 | 7.9±0.5 | 471.6±19.6 | 50.7±3.7 | 20.0±0.5 | 429.0±23.9 |
| Fenced | 46.4±5.7 | 1.16±0.06 | 6.8±0.5 | 39.3±6.2 | 19.0±1.1 | 25.5±1.2 | 8.8±0.5 | 474.9±22.0 | 49.5±3.0 | 19.7±0.6 | 442.9±22.3 |


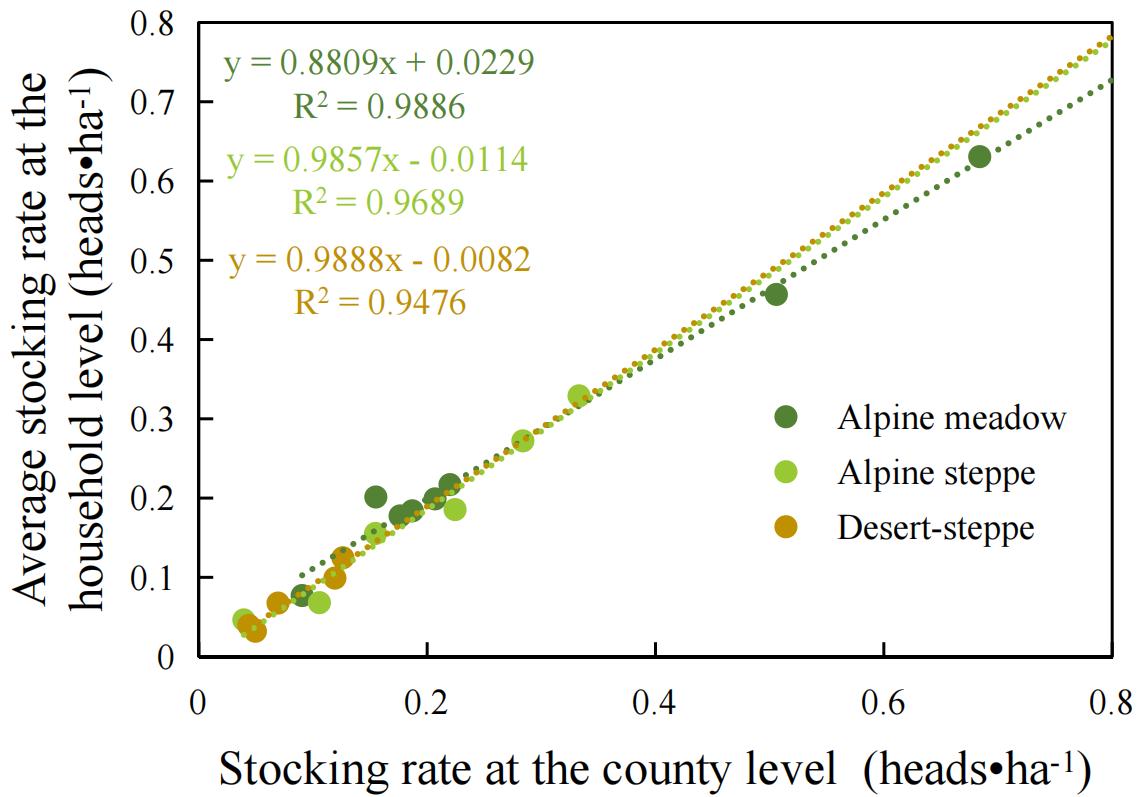


**Figure S1** Linear regressions between stocking rates estimated from statistical yearbook and household livestock rates from the face-to-face interviews with household heads around our sites in 2019.


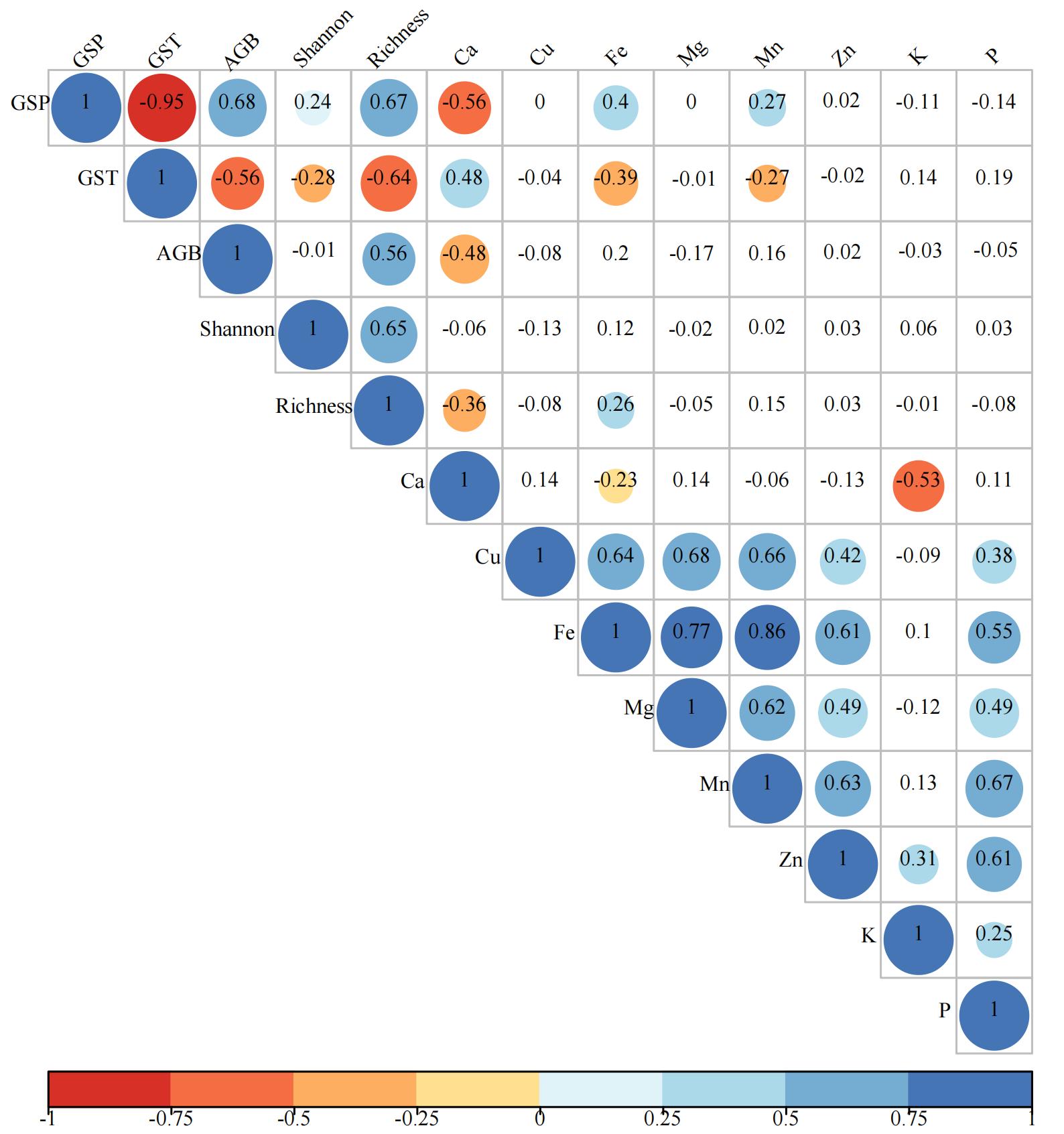


**Figure S2** Correlation matrix of climatic variables (GSP and GST), plant community regimes (AGB, species richness, and Shannon-Wiener index), and soil mineral elements (Ca, Cu, Fe, Mg, Mn, Zn, K, and P) across AM, AS, and DS, North Tibet of China. The colored solid circles represent the significant correlation (*P* < 0.05).


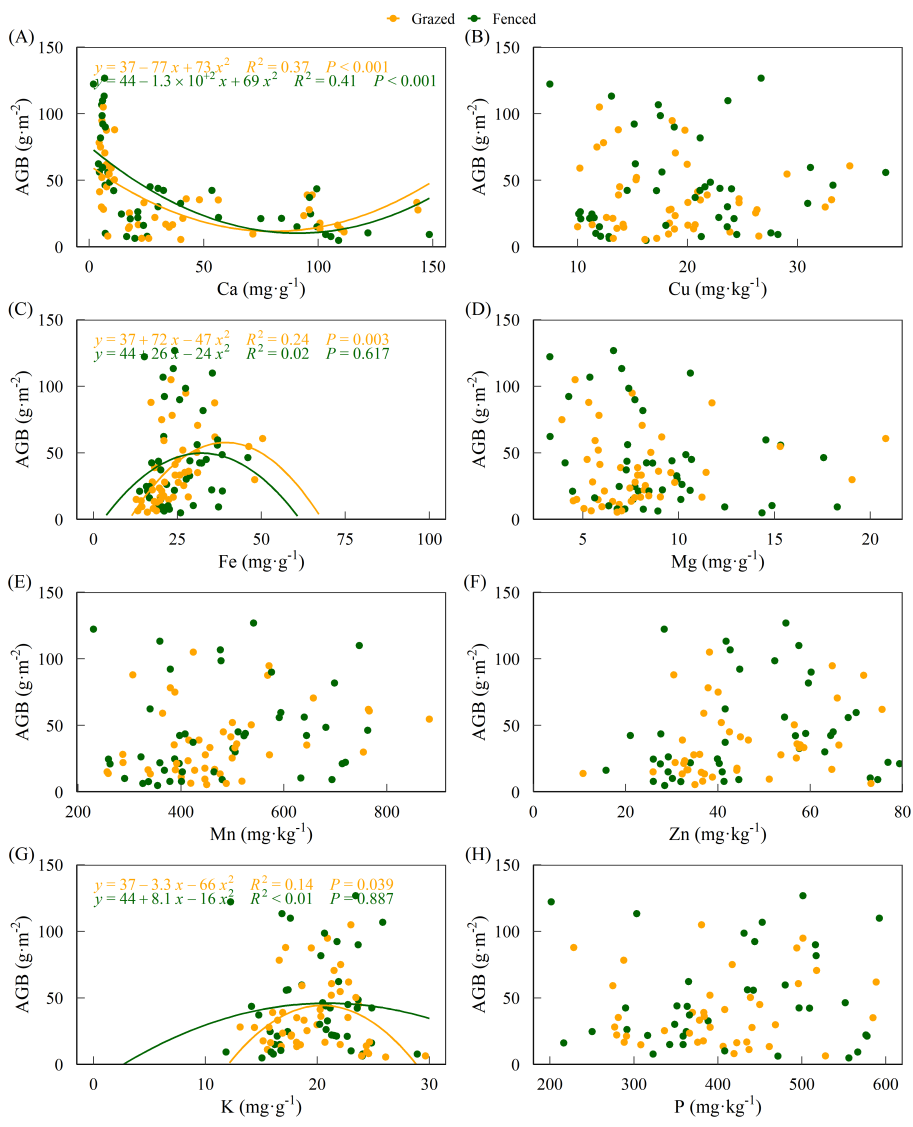


**Figure S3** The relationships of plant aboveground biomass (AGB) with soil mineral elements between fenced and grazed sites across North Tibet, China.


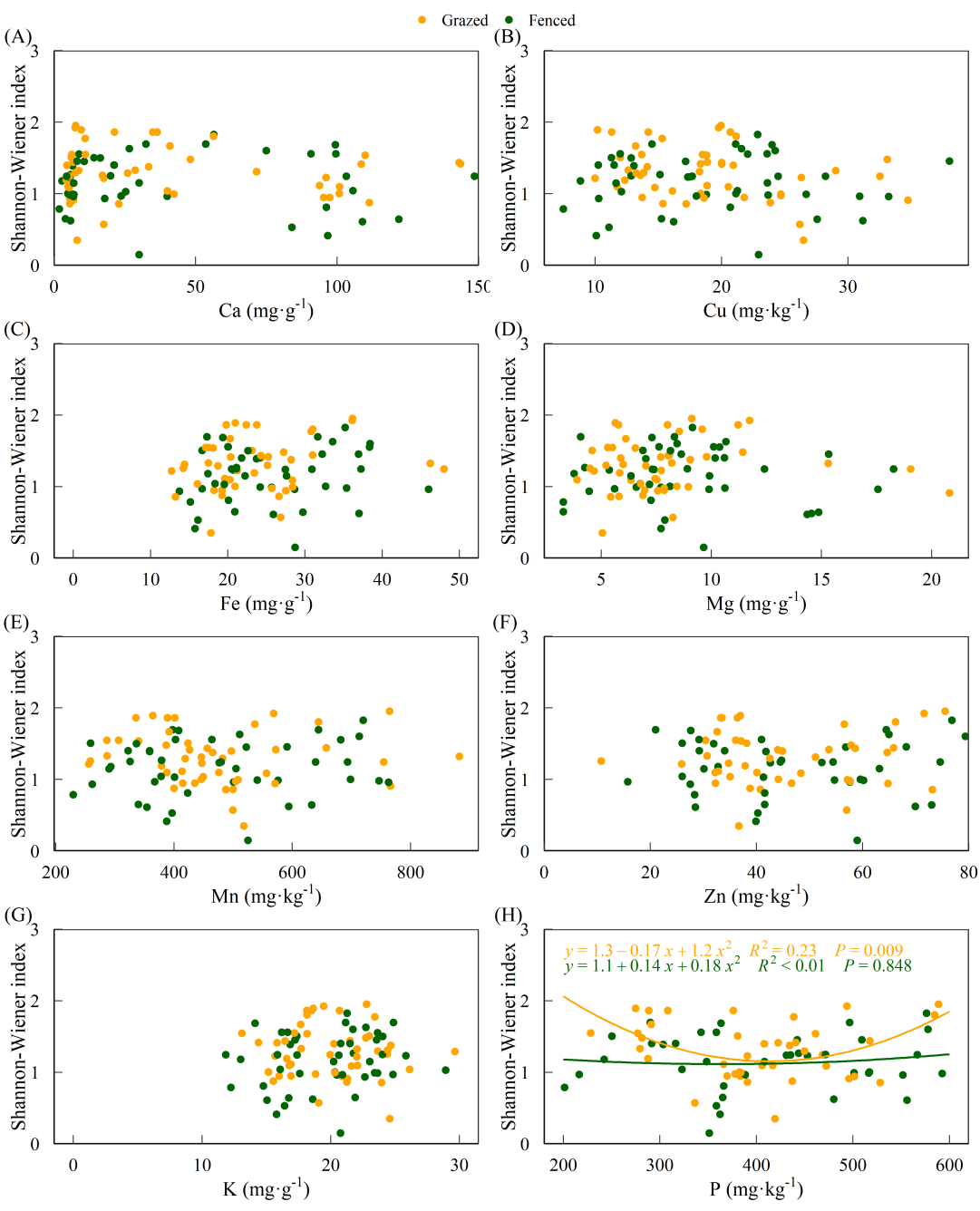


**Figure S4** The relationships of Shannon-Wiener index with soil mineral elements between fenced and grazed sites across North Tibet, China


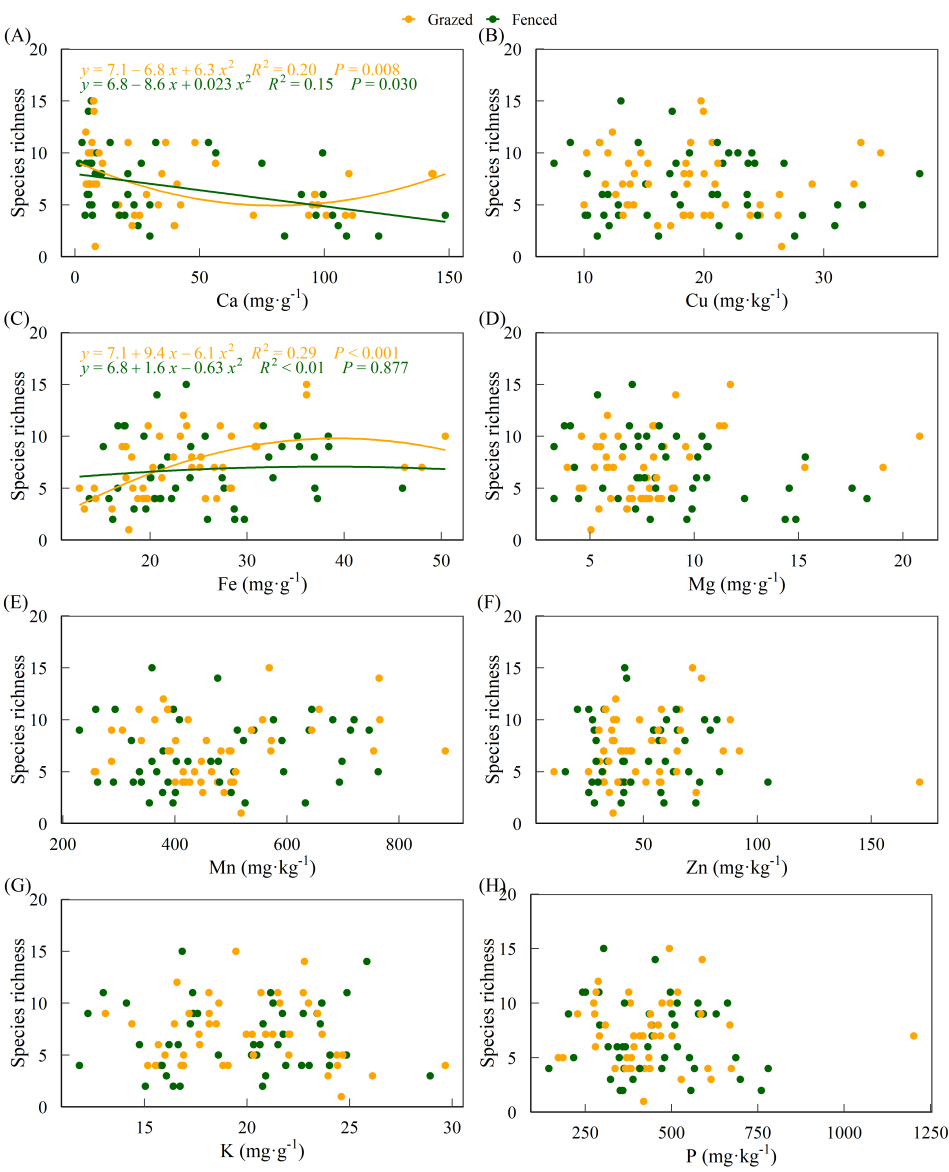


**Figure S5** The relationships of species richness with soil mineral elements between fenced and grazed sites across North Tibet, China.


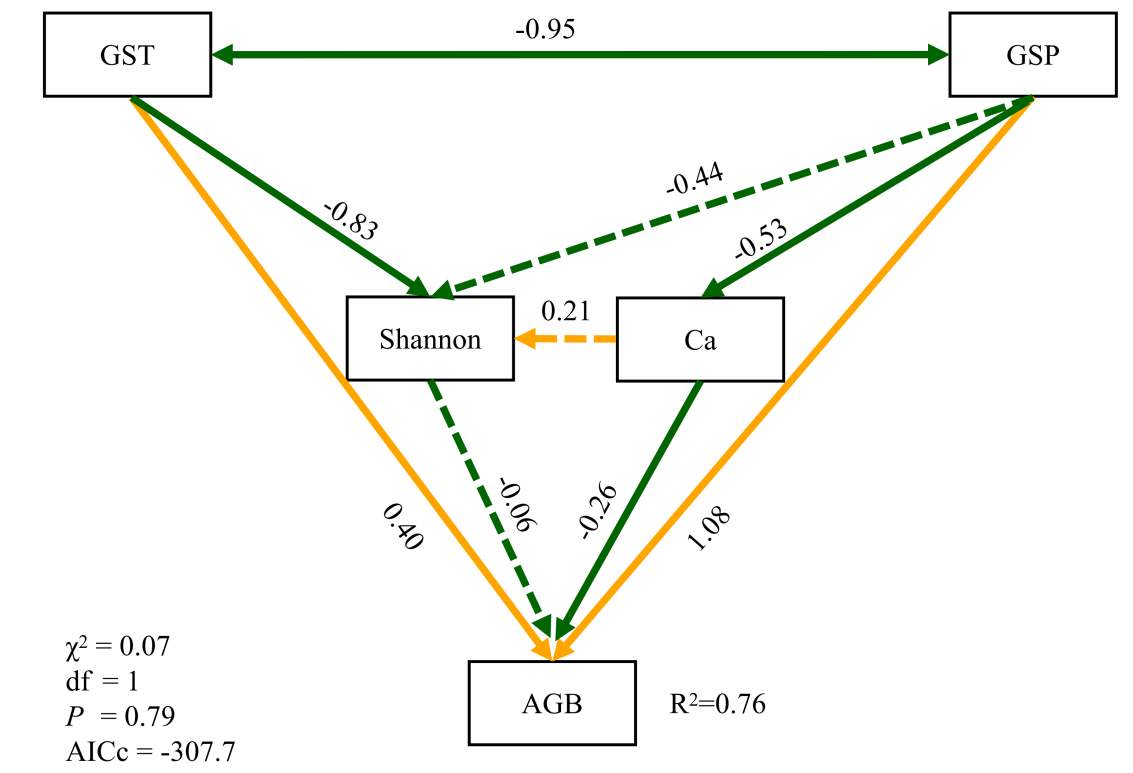


**Figure S6** Structural equation models. Dark-green and orange arrows, respectively, indicate the negative and positive associations. Solid and dashed lines, respectively, indicate the significant and non-significant effects at the 0.05 level.


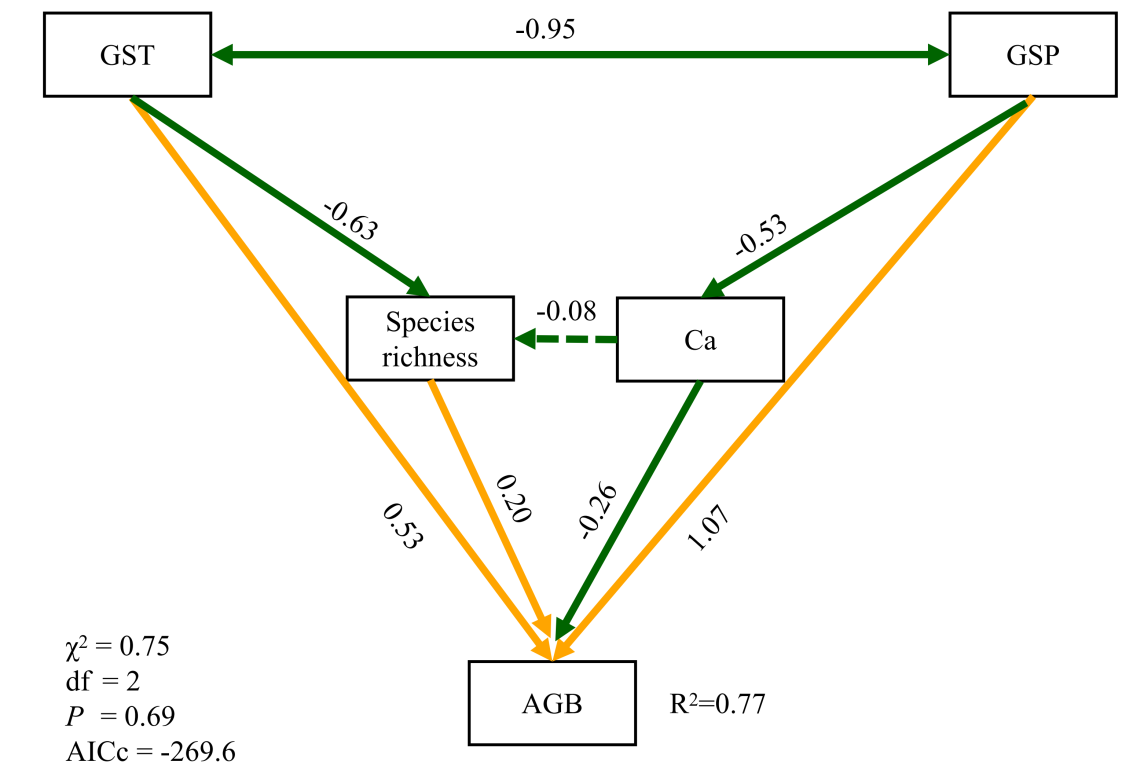


**Figure S7** Structural equation models. Dark-green and orange arrows, respectively, indicate the negative and positive associations. Solid and dashed lines, respectively, indicate the significant and non-significant effects at the 0.05 level.
